# Increased red blood cell distribution width in patients with plaque psoriasis

**DOI:** 10.5937/jomb0-27237

**Published:** 2021-03-12

**Authors:** Paolo Gisondi, Davide Geat, Giuseppe Lippi, Martina Montagnana, Giampiero Girolomoni

**Affiliations:** 1 University of Verona, Department of Medicine, Section of Dermatology, Verona, Italy; 2 University of Verona, Section of Clinical Biochemistry, Verona, Italy

**Keywords:** psoriasis, psoriatic arthritis, RDW, psorijaza, psorijazni artritis, RDW

## Abstract

**Background:**

Red blood cell distribution width (RDW) is frequently increased in inflammatory disorders, and the magnitude of its elevation correlates with disease severity. This study was hence aimed to explore RDW values in patients with psoriasis.

**Methods:**

The study population consisted of 366 adult patients with mild to severe plaque psoriasis and 366 age- and sex-matched blood donor controls. For each psoriatic patient, demographic, clinical, and laboratory data were regularly collected.

**Results:**

RDW and MCV were significantly higher in psoriatic patients compared to controls (13.95 vs. 13.40% and 90.4 vs. 89 fL; both p<0.01). In order to assess whether RDW elevations were related to psoriasis severity, we divided our psoriatic patient population into two groups based on a PASI cut-off of 10. No significant differences were observed between the two groups (i.e., PASI>10 and 10) in terms of RDW (p=0.36). Adopting different PASI cut-offs (i.e. 3, 5, 7, 12) did not result in statistically significant differences (p=0.93, 0.48, 0.22, 0.42, respectively). In linear regression analysis, no significant correlation was found between RDW and PASI or CRP, nor with age, gender, or the psoriasis comorbidities listed in Table I. Furthermore, no significant difference in RDW values was noted between psoriatic patients with and without PsA (p=0.27).

**Conclusions:**

The results of this study confirm that RDW is elevated in psoriatic patients, though the magnitude of its increase did not appear to be associated with disease severity.

## Abstract (sr)

**Uvod:**

Širina distribucije eritrocita (RDW) se često povećava kod zapaljenskih poremećaja i razmere povećanja su u korelaciji sa težinom bolesti. Cilj ove studije je bio da istraži vrednosti RDW kod pacijenata sa psorijazom.

**Metode:**

Populacija ispitanika je bila sačinjena od 366 odraslih pacijenata sa blagom do teškom psorijazom plaka i 366 kontrolnih davalaca krvi usklađenih po pitanju godina i pola. Za svakog psorijatičnog pacijenta redovno su se prikupljali demografski, klinički i laboratorijski podaci.

**Rezultati:**

RDW i MCV su bili značajno veći kod psorijatičnih u poređenju sa kontrolnim pacijentima (13,95% prema 13,40% i 90,4 prema 89 fL; oba p<0,01). Da bismo procenili da li su povišenja RDW povezana sa težinom psorijaze, podelili smo populaciju psorijatičnih pacijenata u dve grupe na osnovu PASI vrednosti od 10. Nisu primećene značajne razlike između dve grupe (tj. PASI>10 i 10) u smislu RDW (p=0,36). Usvajanje različitih PASI graničnih vrednosti (tj. 3, 5, 7, 12) takođe nije rezultiralo statistički značajnim razlikama (p=0,93, 0,48, 0,22, 0,42, respektivno). U linearnoj regresionoj analizi takođe nije pronađena značajna korelacija između RDW i PASI ili CRP, kao ni u vezi sa godinama, polom ili komorbi ditetima psorijaze navedenim u Tabeli I. Pored toga, nije zabeležena značajna razlika vrednosti RDW između psorijatičnih pacijenata sa i bez PsA (p=0,27).

**Zaključak:**

Rezultati ove studije potvrđuju da je RDW povišen kod psorijatičnih pacijenata, iako se čini da veličina njegovog povećanja nije povezana sa težinom bolesti.

## Introduction

Red cell distribution width (RDW) is a reliable marker of red cell size variability (i.e., anisocytosis), calculated as the standard deviation of red blood cell volume/mean corpuscular volume (MCV) ×100. Besides anemia, RDW was found to be increased in several conditions, including inflammatory disorders such as rheumatoid arthritis and inflammatory bowel diseases, cardiovascular diseases (e.g., acute coronary syndromes, heart failure, atrial fibrillation, stroke, and venous thromboembolism), diabetes, cancer, infections, liver and kidney diseases [1]. In particular, increased RDW has been associated with cardiovascular risk and mortality [2]. In recent years, RDW elevation has also been described in patients with psoriasis and has been associated with psoriasis severity and with biomarkers of inflammation such as C reactive protein (CRP) [3] [4] [5].

We carried out a retrospective observational study aimed at assessing RDW values in psoriatic patients and its association with psoriasis severity and C reactive protein (CRP). The study included 366 adult patients with mild to severe plaque psoriasis followed at the Dermatology Unit of the University Hospital of Verona between January and May 2020, and 366 age- and sex-matched controls, i.e., blood donors. The exclusion criterion for psoriatic patients was any topical or systemic treatment/phototherapy for the previous 1 or 3 months, respectively. Moreover, subjects with hematologic disorders, hemoglobin (Hb) levels < 120 g/L in women and < 130 g/L in men, malnutrition, atrial fibrillation, heart failure, acute venous thromboembolism, malignancy, immunodeficiencies, active infections, inflammatory diseases other than psoriasis, hepatic or renal disease, and pregnant women were excluded. For each psoriatic patient, demographic data, body mass index, Psoriasis Area Severity Index (PASI), psoriasis duration, and the following comorbidities: psoriatic arthritis (PsA), overweight or obesity, hypertension, type 2 diabetes mellitus, dyslipidemia (i.e., hypercholesterolemia and/or hypertriglyceridemia) and previous cardiovascular events (defined as ischemic heart disease, peripheral arterial disease, stroke/transient ischemic attack, or revascularization procedure) were registered. The following laboratory parameters were collected: hemoglobin (Hb), mean corpuscular volume (MCV), RDWand CRP. Statistical analysis was performed with STATA statistical software. Data were tested for normality using the Kolmogorov-Smirnov test. Student t-test and chi-square test were used to compare normal continuous and categorical variables in cases and controls, respectively. Multiple linear regression and Pearson's correlation were performed to test possible associations between RDW and clinical variables in psoriatic patients.

Patient characteristics are shown in Table 1. RDW and MCV were significantly higher in psoriatic patients compared to controls (13.95±1.52 vs. 13.40±0.57% and 90.4±5.7 vs. 89.0±7.1 fL, respectively). In order to assess whether RDW elevations were related to psoriasis severity, we divided our psoriatic patient population into two groups based on a PASI cut-off of 10. No significant differences were observed between the two groups (i.e., PASI > 10 and ≤ 10) in terms of RDW (p=0.36). Adopting different PASI cut-offs (i.e. 3, 5, 7, 12), did also not result in statistically significant differences (p=0.93, 0.48, 0.22, 0.42, respectively). A PASI cut-off of 10 was initially used as this is the most widely accepted cut-off between mild and moderate-to-severe psoriasis [6]. Other cut-offs, however, are reported in the literature ⎯ such as PASI < 7 defining mild psoriasis and > 12 defining severe psoriasis [7]. Lastly, PASI scores of 3 and 5 have also been used as target outcomes [8]. In linear regression analysis, no significant correlation was found between RDW and PASI or CRP, nor with age, gender, or the psoriasis comorbidities listed in Table 1. Furthermore, no significant difference in RDW values was noted between psoriatic patients with and without PsA (p=0.27).

**Table 1:** Characteristics of the study population. Hb, hemoglobin; RDW, red cell distribution width; MCV, mean corpuscular volume; CRP, C-reactive protein; PASI, Psoriasis Area and Severity Index; CV, cardiovascular.

	Psoriatic patients (n=366)	Controls (n=366)	P-value
Males (%)	61.2	61.2	1
Age (y)	55.7 ± 13.9	55.5 ± 13.8	0.8
Hb (g/l)	143.3 ± 13.2	144.4 ± 12.4	0.7
RDW (%)	13.95 ± 1.52	13.40 ± 0.57	0.001
MCV (fl)	90.40 ± 5.70	89.04 ± 7.13	0.003
CRP (mg/l)	3.91 ± 1.87	3.41 ± 2.01	0.6
Psoriasis duration (y)	16.7 ± 10.7	-	
PASI	12.4 ± 10.4	-	
**Comorbidities (%):**			
Overweight or obesity	49.2	24.5	0.001
Psoriatic arthritis	31.3	-	
Hypertension	44.3	19.5	0.001
Type 2 diabetes mellitus	15.9	3.8	0.001
Dyslipidemia	21.3	12.6	0.001
Previous CV events	12.3	8.1	0.01

The elevation in RDW and MCV in psoriatic patients found in our study is coherent with previous studies, though we failed to find a correlation with psoriasis severity, CRP, or with the presence of PsA. RDW elevation in plaque psoriasis was first described by Kim et al. [5] in 2015, who proposed this as a marker of psoriasis-associated systemic inflammation. Indeed, systemic inflammation leads to more significant oxidative stress, damage of circulating red blood cells, and inhibition of erythropoiesis with impaired erythrocyte maturation [9] [10] [11]. Furthermore, proinflammatory cytokines were also shown to alter iron metabolism, which is a crucial factor for erythropoiesis.

In conclusion, albeit we can confirm that RDW values are increased in patients with psoriasis, our findings do not support its routine assessment for predicting or monitoring disease severity. Additional and more extensive studies would be needed at this point to establish whether RDW or not might be a biomarker of inflammation in patients with psoriasis that is not reflected by the PASI score or CRP.

## List of abbreviations

RDW, red cell distribution width; MCV, mean corpuscular volume; CRP, C-reactive protein; Hb, hemoglobin; PASI, Psoriasis Area Severity Index; PsA, psoriatic arthritis.

## Funding

None.

## Conflict of interest statement

The authors stated that they have no conflicts of interest regarding the publication of this article.
